# The Application of Critical Power, the Work Capacity above Critical Power (W′), and Its Reconstitution: A Narrative Review of Current Evidence and Implications for Cycling Training Prescription

**DOI:** 10.3390/sports8090123

**Published:** 2020-09-04

**Authors:** Alan Chorley, Kevin L. Lamb

**Affiliations:** Department of Sport and Exercise Sciences, University of Chester, Parkgate Road, Chester CH1 4BJ, UK; k.lamb@chester.ac.uk

**Keywords:** critical power, W′, reconstitution, cycling, endurance, training, severe intensity

## Abstract

The two-parameter critical power (CP) model is a robust mathematical interpretation of the power–duration relationship, with CP being the rate associated with the maximal aerobic steady state, and W′ the fixed amount of tolerable work above CP available without any recovery. The aim of this narrative review is to describe the CP concept and the methodologies used to assess it, and to summarize the research applying it to intermittent cycle training techniques. CP and W′ are traditionally assessed using a number of constant work rate cycling tests spread over several days. Alternatively, both the 3-min all-out and ramp all-out protocols provide valid measurements of CP and W′ from a single test, thereby enhancing their suitability to athletes and likely reducing errors associated with the assumptions of the CP model. As CP represents the physiological landmark that is the boundary between heavy and severe intensity domains, it presents several advantages over the de facto arbitrarily defined functional threshold power as the basis for cycle training prescription at intensities up to CP. For intensities above CP, precise prescription is not possible based solely on aerobic measures; however, the addition of the W′ parameter does facilitate the prescription of individualized training intensities and durations within the severe intensity domain. Modelling of W′ reconstitution extends this application, although more research is needed to identify the individual parameters that govern W′ reconstitution rates and their kinetics.

## 1. Introduction

The hyperbolic relationship between exercise intensity and duration in humans has long been established [1], however, it was not until the advent of the critical power (CP) concept [2], that a simple mathematical framework (Equation (1)) for the power–duration curve was devised based on the two parameters of a fixed energy reserve and a maximum rate of energy reconstitution:

W_lim_ = A + B × T_lim_ (1)

where W_lim_ = limit of work (J); A = fixed energy reserve (J); B = critical power (W); T_lim_ = time limit (s).

The concept was successfully applied to cycling [3] and, importantly, CP was found to be highly correlated to the “anaerobic threshold”, whilst the sum of CP and the fixed energy reserve was highly correlated to maximal oxygen uptake ($\dot{V}O$_2max_). The correlations provided a link between the parameters of the mathematical model and the underlying physiology, suggesting that exercise intensities above CP deplete the fixed energy reserves owing to an insufficient supply of oxygen for aerobic metabolism. Poole, et al. [4], provided further evidence that CP did indeed represent a physiological threshold by demonstrating that intensities above CP did not evoke a $\dot{V}O$_2_ steady state. In this “severe intensity domain” $\dot{V}O$_2_ continued to rise until $\dot{V}O$_2max_ was attained and exhaustion ensued, whilst “heavy intensity domain” exercise performed slightly below CP resulted in the attainment of a $\dot{V}O$_2_ steady state without exhaustion. Despite the simplicity of the CP model and the association of CP with a physiological threshold of sustainable exercise, other measures of aerobic capacity have received arguably more research attention. Lactate turn-point [5], lactate threshold [6], onset of blood lactate accumulation [7], maximal lactate steady state (MLSS) [8], respiratory compensation point [9,10] and ventilatory threshold [6], are all intended to identify or approximate an anaerobic threshold or maximum intensity of steady state exercise through various invasive (blood lactate) and non-invasive (pulmonary gas analysis) procedures. A variety of procedures exist to determine these measures, but most (apart from those to determine MLSS) can be completed within one or two laboratory visits. CP testing, however, requires between two and seven laboratory visits to produce a valid measurement of CP [11], hence it is likely such a demanding schedule of exhaustive tests has impeded the widespread adoption of CP testing, particularly by athletes and their coaches. The advent of the 3-min all-out test [12], was perhaps the catalyst for an upsurge in research investigating and utilizing critical power. The protocol simply required a subject to pedal as fast as possible for the entirety of the three minutes. CP could be interpreted as the levelling of aerobically driven power output during the last 30 s of the test after all anaerobic work or W′ had been spent earlier in the effort. W′ could then be calculated as the total amount of work done above CP during the three minutes. Whilst the test protocol did require a preliminary laboratory visit to tune the test parameters, it offered the physiological and performance-related measures of critical power and W′ in a much less demanding manner than before.

Whilst CP is arguably the “best” measure of a maximum steady state of predominantly aerobic metabolism [13], and as such demarcates the severe and heavy intensity domains, and is strongly correlated to muscle capillarity [14], the underlying physiology of W′ remains uncertain. Originally the parameter was described as a fixed energy reserve [2], or anaerobic work capacity [15], dependent upon oxygen stores within the muscle, high energy phosphates and anaerobic glycolysis [3,16]. That W′ is entirely anaerobic and independent of external oxygen availability is questionable following evidence of a reduction in W′ at high altitude [17], and a reduction in W′ in hypoxia, with the latter being correlated to a reduced delta between $\dot{V}O$_2_ at CP and $\dot{V}O$_2max_ [18]. Moreover, the magnitude of $\dot{V}O$_2max_ and the development of the $\dot{V}O$_2_ “slow component” within the severe intensity domain have been shown to be determinants of W′ [19]. Latterly, W′ has been associated with the accumulation of fatiguing metabolites such as inorganic phosphates and hydrogen ions [20,21,22], with W′ in a cycling test shown to be reduced immediately following severe upper body exercise, presumably owing to the transport or accumulation of metabolites in the bloodstream [23]. Despite the lack of a clear understanding of the bioenergetics of W′, the parameter continues to be a good predictor of work capacity above CP.

CP, W′ and the reconstitution of W′ have become recognized as important physiological indices within sports science, yet they have not been widely adopted by coaches and athletes within cycle sport. Therefore, the aim of this narrative review is to explore the CP concept from the perspective of its application into cycle sport. Specifically, the review focuses on testing methodologies, the application of W′ reconstitution to intermittent exercise and the applicability to cycle training prescription.

## 2. Assumptions of the Critical Power Model

As the CP model is a simple mathematical interpretation of numerous complex physiological activities within the body, it has inherent limitations based upon several important “assumptions” [24,25]. Such assumptions yield potential sources of error in the model which need to be eliminated or minimized within a test protocol. These assumptions include:

1. The aerobic supply of energy is unlimited for any duration.
2. Cycling efficiency remains constant.
3. Power output is limited solely by duration and tends towards infinity as time duration approaches 0 s.
4. All power output demands up to CP are immediately and constantly fulfilled by aerobic mechanisms up to that limit.
5. At exhaustion W′ is fully depleted, i.e., W′ equals 0 J.

Whilst CP was originally described as being sustainable for “a very long time” [2], studies investigating time to exhaustion (TTE) at critical power have observed sustainable durations varying from 20–40 min [26], to over an hour [27]. TTE trials at CP have also been shown to be susceptible to a learning effect [27], seemingly questioning the meaning of exhaustion. Recently, the effects on CP after prolonged bouts of heavy intensity cycling demonstrated that durations beyond 80 min resulted in a reduction of CP, but with carbohydrate ingestion CP is maintained beyond 120 min. The same carbohydrate intake however, did not preserve W′ which began to reduce after 40 min of cycling in both conditions due to unspecified fatigue processes [28]. The researchers associated this reduction in CP (owing to reduced glycogen availability) with a reduction in cycling efficiency from 80 min onwards due to a shift from carbohydrate oxidation to fat oxidation with its associated higher metabolic cost [29].

That the duration at which CP can be maintained is somewhat unpredictable should be expected; it represents the physiological boundary between heavy and severe intensity domains but does not consider the complexities of fatigue, motivation, and substrate availability. At the opposite end of the duration scale, as the time asymptote approaches zero in the two-parameter model instantaneous power output tends towards infinity. In an attempt to address this, a three-parameter model was developed [30,31], whereby maximum power output is proportional to the remaining anaerobic work capacity. However, as the three-parameter model has been shown to produce comparatively low values of CP and questionably high values of W′ [32,33], either owing to the disputed validity of the CP and W′ estimations, or simply because of a lack of a need for a CP model to cover very short time durations, the two-parameter model remains the favoured model for research purposes.

The assumption that power output is instantaneously met by aerobic metabolism up to the CP ceiling is a source of error [34]. During test protocols and actual cycling, oxidative metabolism will encounter a delay responding to increases in the power output demands, with the shortfall being met by anaerobic metabolism causing a partial depletion of W′. The amount of W′ expended during responses to such increases in power will vary between individuals and can be manipulated by external factors such as prior exercise [35]. Accordingly, test protocols should seek to minimise the utilization of W′ that occurs because of such delays in the aerobic response.

Accepting that W′ is fully expended at the point of volitional exhaustion has been a necessary assumption to enable the calculations for CP and W′; W′ can only be determined by knowing the total amount of work done during a test session and, thereby, the anchoring of exhaustion to a completely expended W′. With the current scientific understanding of what constitutes W′ it remains unknown whether exhaustion really does occur when W′ equals 0 J; however, it is imperative that test subjects consistently endeavour to expend the W′ available to them to yield valid measurements of W′. Interestingly, many studies have simply implied full depletion of W′ by using terms such as the “participants were highly motivated” [36,37,38] or “were provided with strong verbal encouragement” [39,40]. Whilst this may provide a consistency in the test procedures, such words cannot eradicate subjective influences on test performance. The reality is that cycling at intensities above CP is an uncomfortable (“painful”) experience, especially as the point of exhaustion approaches, so the urge to stop must be overcome until the legs simply cannot turn the pedals any longer. Indeed, the psychological aspects of exercise tolerance (effort perception [41], mental fatigue [42], unpleasantness and pain perception [43]) have been explored and it is proposed that often participants will end exercise not owing to physiological limitations, but rather to an unwillingness to continue, and that such psychological effects lead to task disengagement [41]. Furthermore, the reliability of testing to exhaustion is susceptible to learning effects [27,44], participant training status [45], and central fatigue [42,43]. Therefore, the assumption of full W′ depletion cannot be warranted by any protocol due to the lack of direct W′ measurement techniques. Therefore, it is important that methods for determining CP and W′ look to ensure that W′ is as close as possible to full depletion. As such, consideration should be given to ensuring the attainment of $\dot{V}O$_2max_ at the end of exercise, which is a pre-requisite for the complete expenditure of W′ [46,47], minimizing the amount of work completed whilst aerobic metabolism is less than CP, and reducing the opportunity for negative psychological consequences.

## 3. Methods for Determining Critical Power and W′

**Figure 1:**
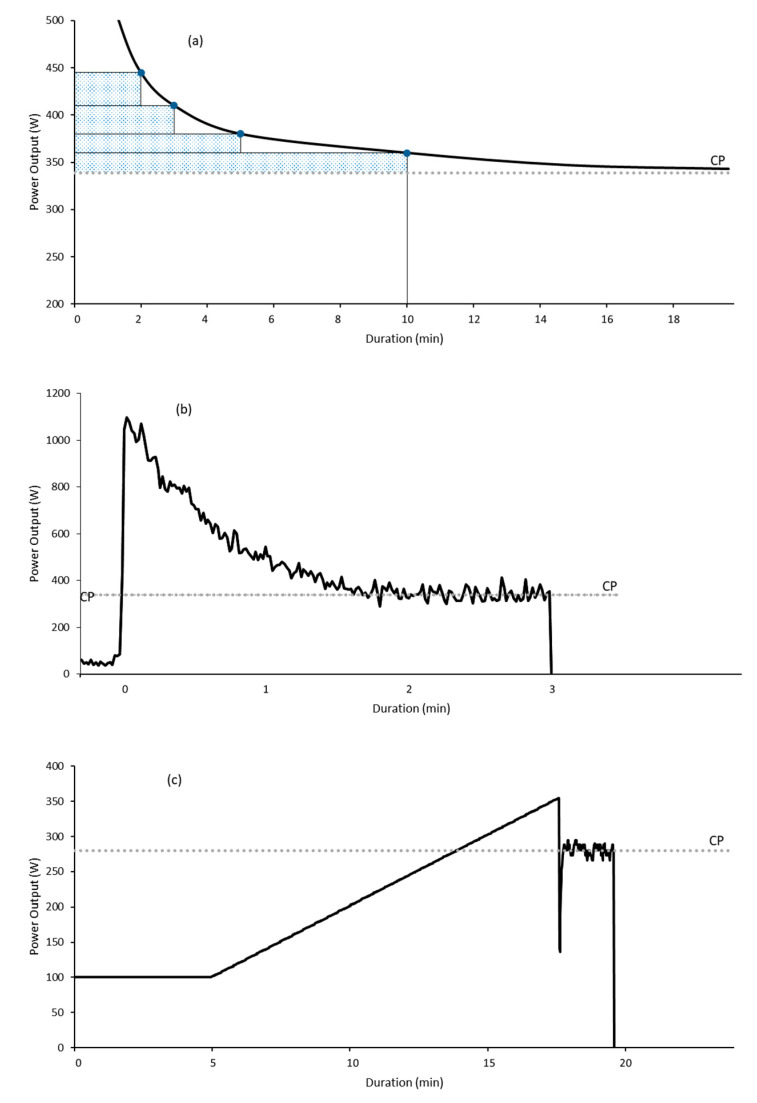
Typical power profiles of tests to determine critical power (CP) and W′ (the work capacity above CP). (**a**) Constant work rate tests to estimate CP as the asymptote of the power-duration relationship, and W′ as the area of the shaded rectangles above CP. (**b**) 3-min all-out test estimating CP as the average power of the final 30 s and W′ as the total work done above CP. (**c**) Ramp all-out test estimating CP to as the average power of the final 30 s of the all-out phase, and W′ as the total work done above CP.

Currently there are three distinct test methodologies [2,12,48] (see Figure 1) used to determine CP and W′, all requiring exercise to exhaustion, as such they are extremely demanding on the athlete and protocols continue to evolve to lessen that burden. 

### 3.1. Constant Work Rate Tests

Constant work rate (CWR) tests have been the criterion against which other tests protocols have been measured [48,49], and remain popular for research studies [14,42,50,51,52]. Following a preliminary ramp test to estimate critical power the participants undertake typically three, but as many as seven, TTE trials at differing intensities above CP intended to elicit exhaustion after 2–15 min [22,53]. Three mathematical derivations exist of plotting the power-duration data in order to determine CP and W′; non-linear power-time (Equation (2)), linear work-time (Equation (3)) and linear power-time^−1^ (Equation (4)).

T_lim_ = W′/(P − CP) (2)

W = CP × T_lim_ + W′ (3)

P = W′ (1/T_lim_) + CP (4)

where W = total work done (J); W′ = finite amount of work above CP (J); CP = critical power (W); T_lim_ = time limit (s); P = power output (W).

The three models should produce the same measurements of CP and W′ if all trials are truly completed to exhaustion and a perfect fit is obtained. However, it is argued that, where the perfect fit does not exist, the model producing the smallest standard error of estimate be used [52,54,55,56]. Whilst aligning the trial data to a best fit may model may produce statistically sound results, it should be noted that this may not represent the most accurate determination of CP and W′. Although a participant can underperform on any given trial, he/she cannot out-perform their own physiological limits, hence a best fit approach to all the trial data will always underestimate CP, W′ or both.

Perhaps the biggest limitation of the CWR method is that it is fundamentally a series of TTE trials, which have long been the subject of questionable reliability [57], and compare unfavorably to actual time trials [58]. More recently it has been shown that a greater mean work rate is performed using self-paced time trials than corresponding TTE trials resulting in higher estimates of CP from the time-trial protocol [54]. As it is essential for W′ to be fully depleted during every estimation trial in order to obtain valid estimates, these studies together with those showing a learning effect provide evidence that W′ is often not fully depleted [11,44] during TTE trials. The duration of TTE trials appears to influence the depletion of W′. Shorter duration TTE trials have been shown to produce higher estimates of CP and lower W′ than longer trials despite having similar goodness of fit *R*^2^ values [59]. Furthermore, the fit method of the mathematical models results in the derived values of CP being more sensitive to the longer duration trials, and shorter trials having a greater influence on W′. It has also been shown that 30 s all-out efforts immediately following the cessation of TTE trials have yielded an increase of power above CP; a scenario that simply should not be possible after the full depletion of W′ [51]. Furthermore, this study demonstrated that the longer the duration of the TTE the greater the amount of work that could be performed in a subsequent 30 s effort, implying that the longer the TTE the more W′ that is spared. This sparing of W′ particularly over longer trials is likely owing in part to motivational and other psychological factors, which for some time have been a consideration in long TTE trials [60,61]. Perceived exertion has been proposed as the reason for the termination of exercise in TTE trials lasting around 14 min [43], (which is towards the upper end of durations used in CP trials), whilst mentally fatiguing tasks prior to exercise have increased physiological markers during TTE resulting in exercise being terminated earlier [42]. Furthermore, a shift from peripheral fatigue towards central fatigue is found as the duration of exercise increases [62]. As such it is reasonable to assume that the longer the TTE duration the further from complete expenditure of W′ the participant is at the premature termination of exercise. Moreover, given such a scenario the best fit methods would lead to an underestimation of CP and potential overestimation of W′. Validation of laboratory-based CP and W′ estimations from TTE trials against similar field measures comprising self-paced, fixed-duration cycling efforts performed at an outdoor velodrome has established statistical agreement via negligible bias and low within-subject variability of CP values, but not W′, which was on average approximately 5 kJ higher in the field tests [63], indicating a greater amount of work performed.

As alluded to above, the utility of CWR testing for athletes is severely impacted by the number of laboratory visits required. Whilst an estimation of CP may be obtained from training data and may negate the need for the preliminary visit [45], an assessment would still require a minimum of three visits on separate days and control exerted over prior training. Thus, the detrimental impact on an athlete’s training plan is likely to be unappealing. Alternatively, researchers have sought to complete multiple TTE trials (with 30 min and 3 h recoveries) within a single day [64], but when validated against multi-day testing (with 24 h recovery) W′ values were underestimated suggesting an incomplete reconstitution of W′. [65]. Likewise, comparisons of single day laboratory TTE trials against “best” training and race data of the same durations (3, 7 and 12 min) over a five-week period [66] yielded significant underestimations of W′ from the laboratory TTE trials. This was despite the likelihood that such race efforts themselves would not result in the full expenditure of W′ due to the data from the actual efforts being clipped to match the test durations, and that the nature of a race would prevent the limit of tolerance being reached owing to the necessity to continue racing.

### 3.2. Three-Minute All-Out Test 

Referred to previously, the 3-min all-out test is a single test to establish both CP and W′ parameters. However, a preliminary ramp test is used to determine the mid-way point between the gas exchange threshold (GET) and $\dot{V}O$_2peak_ which is then used as an estimate for CP in the subsequent all-out test undertaken on a separate day. The all-out test comprises a short warm-up followed by a 3-min all-out effort where the participant pedals as fast as possible throughout the duration of the test. The resistance of the ergometer is set such that the estimated CP from the preliminary test is attained at the participant’s preferred cadence. The test works on the premise that W′ is fully expended during the first 2.5 min of the test, leaving the power output of the final 30 s to reflect the CP asymptote. That power output is CP during the final 30 s is mathematically shown by Equation (4). Thus, CP is deemed to be the average power output during the final 30 s and W′ is the total work done above CP during the 3-min effort [12]. When combined with the initial determination of the GET, this provides an athlete with a profile of the both the moderate-heavy and heavy-severe intensity domain boundaries along with W′ capacity. Burnley, et al. [12], validated the test by having the participants cycle above and below the measured CP. At a CWR 15 W above CP, participants failed to achieve a steady $\dot{V}O$_2_ and blood lactate measurements rose inexorably until exhaustion, whilst at 15 W below CP the majority of them completed 30 min achieving a $\dot{V}O$_2_ steady state and stable blood lactate levels. Successful validation of CP and W′ derived from the 3-min test against those derived from CWR tests has been subsequently demonstrated [49,67], however the 3-min all-out test reportedly overestimated CP and underestimated W′ when compared to measures derived from time trials in elite cyclists [36], and in trained cyclists [68,69]. However, in the latter studies actual total work done during the 3-min tests far exceeded what the CWR tests would have predicted as possible, suggesting the CWR-derived estimates of CP were low. Moreover, the Bartram, et al. [36], study undertook the CWR tests during a single day without accounting for the incomplete reconstitution of W′ seen within single-day testing [65].

Although the all-out test lasts only three minutes, the discomfort the participant suffers should not underestimated. Consequently, a high number of invalid tests have reported due to factors such as: failure to complete the test duration, power output prematurely dropping below end power and allowing recovery of W′, and power output failing to plateau during the final 30 s [70]. Despite the difficulties of test execution the 3-min all-out test has been shown to be robust to pacing strategies in the first 30 s of the test so long as W′ continues to fall and is fully spent prior to the plateau of power output during the final 30 s [40,71]. However, an appropriate prior estimation of CP and preferred cadence are essential for valid data as an end cadence just 10 r∙min^−1^ greater than that preferred has been shown to significantly reduce the CP measurement derived from the test. The 3-min all-out test relies heavily on the assumption that aerobic metabolism instantly provides energy up to the individual’s CP. That power output peaks within the first 5–10 s of the test [49,68], yet $\dot{V}O$_2_ does not peak until approximately 80 s into the test [12], suggests that the contribution of aerobic metabolism during the first half of the test does not meet the demand hence there is a greater reliance upon anaerobic metabolism, resulting in a possible underestimation of W′. Conversely, during the last 30–60 s of the test where power output seemingly plateaus, small variations in power output above and below CP are observed [69], yielding a small increase to the measured W′ due to the additional work done above CP.

It is possible that due to the demanding nature of the 30-min all-out test many studies have also included a familiarisation visit [12,49,67,72], although arguably this could be appended to the preliminary visit as some researchers have done [68,69]. To further reduce the time burden of CP testing, the preliminary ramp and 3-min all-out tests have been combined in to a single day of testing with just 20 min of recovery between the ramp test and 3-min all-out test [73]. However, given that W′ has been shown to not fully recover within such short periods, the data may be questionable [65]. Some studies have sought to negate the need for a preliminary test altogether by setting a resistance on the ergometer based on body mass [70,74,75,76], but given the sensitivity of the test to variations away from preferred cadence, such population-based estimations are not likely to find favour with athletes or researchers requiring precision in their results. Interestingly, given that most trained cyclists now have power and cadence data from training and racing readily available, no studies appear to have based the ergometer resistance on estimates obtained from individual data.

### 3.3. Ramp All-Out Test

Substituting a series of ramp tests of differing ramp rates in place of a series of CWR tests was proposed as a method for the determination of CP and W′ that reduces the motivational burden of completing CWR tests, particularly those of longer duration at power outputs little more than CP [60,61]. However, as mean derived values of CP and W′ were not different between ramp and CWR protocols and the number of tests required remained the same, no tangible advantage over the traditional CWR tests was demonstrated. The ramp format was revisited in the light of the 3-min all-out test, this time using the ramp to fully deplete W′ immediately before switching to a 3-min all-out phase to determine CP [48]. CP could be determined from the power output plateau at the end of the 3-min whilst W′ was calculated as the total work done above CP during the ramp and all-out phases. The resultant value of CP was validated with CWR exercise 10 W above and below CP. All participants completed the validation trial 10 W below CP, and only one completed 30 min above CP, although it was noted that $\dot{V}O$_2_ never attained a steady state. Within the same study measurements of CP and W′ were also compared to those derived from traditional CWR tests, with no mean difference observed between CP, but W′ was significantly smaller in the ramp all-out test. Similarly to CWR and 3-min all-out tests, the ramp test violates the CP model assumption that aerobic metabolism immediately supplies energy up to the limit of CP, however the shallow 20 W∙min^−1^ ramp slope employed would likely mean a small but continuous depletion of W′ throughout the ramp up to the point around CP, owing to the power output demand increasing and aerobic metabolism responding to the increased demand. Whilst this lag can be described in terms of oxygen kinetics [77,78], the extent of any effect on the measured value of W′ remains difficult to quantify, although in comparison to CWR tests a shortfall of approximately 2.5 kJ in total work done during the ramp has been reported, albeit at a much steeper ramp rate, which the authors attribute to the differences in W′ [79].

Examination of the power output data from Murgatroyd, et al. [48], reveals that the transition from ramp to all-out phases, although instant, elicited a short recovery from the participants before power quickly rose and plateaued around CP. This micro recovery would allow partial reconstitution of W′, which could artificially inflate CP during the remainder of the 3-min phase. However, the validation against $\dot{V}O$_2_ responses and comparisons to a CWR test (described above) [48], suggest this inflation of CP is small if it exists at all. Power output during the all-out phase did not vary between 30 s and 180 s, allowing the testing burden of this phase to be reduced to 2 min in later studies [45]. Additionally, in keeping with the 3-min all-out test, the all-out phase of the power output during the ramp test all-out phase contained a small variation above and below CP, which was included in the determination of W′, potentially inflating it albeit by a small amount given that W′ was expected to be fully expended during the ramp phase. A later variation of the ramp all-out test [45], did not include work done above CP during the 2-min all-out phase when determining the W′, but included a novel step-down in power output at the end of the ramp to 30 W above estimated CP to accommodate the notion that W′ may not be fully expended at the peak of the ramp because of the maximal power at any instant being limited to a proportion of the remaining W′ [31,79]. Similar observations of additional work performed slightly above CP following the apparent limit of tolerance have previously been observed [51,80,81].

A notable advantage over the 3-min all out test is that gas analysis performed during the ramp could be used to determine the gas exchange threshold [9], thus providing the demarcation of moderate-heavy, and heavy-severe intensity domains together with W′ from a single test. Neither variation of the ramp all-out test [45,48] used a preliminary test to determine estimates of CP and preferred cadence, despite the validity of an all-out test being dependent upon cadence [71]. Instead, they were either relying on an estimate based on body mass [48,82] for untrained participants, or the subjective examination of training data from trained participants [45]. It is noteworthy that readily available commercial power meters are now commonplace amongst trained cyclists with many systems being validated against laboratory ergometers [83,84,85,86], and as such careful examination of recent training and race history should provide estimates of CP and preferred cadence at least as valid as the 50% delta between GET and $\dot{V}O$_2max_ derived from preliminary tests [12,67].

Like all other tests to the limit of tolerance the ramp all-out test is very demanding, but appears to meet the objective of reducing the motivational and psychological limitations associated with TTE trials, and does not have the high number of reported failed tests associated with the 3-min all-out test [70]. That a single test can yield valid CP, W′ and GET measurements suggests that the ramp all-out test may be worthy of further scientific investigation as it appears that its less demanding schedule will be of particular advantage in applied practice.

## 4. W′ Reconstitution

CP and W′ represent important physiological characteristics that can predict performance in the severe intensity domain. Cycle disciplines that take place exclusively within the severe domain are limited, however, to track events such as the individual pursuit (3 km or 4 km), and, depending on the course, some short road time trials, prologues and hill climbs. Beyond these events and aside from sprint and ultra-endurance events, almost all forms of cycle races are stochastic in nature [87,88,89], involving repeated efforts within the severe intensity domain interspersed with varying degrees of exercise and recovery within the moderate and heavy intensity domains. Performance in such races is, therefore, dependent upon not only the absolute capacity of W′ but on its repeated depletion and reconstitution throughout the course of a race. The expenditure of W′ has been shown to be both linear in relation to power output above CP and independent of the rate of utilization [46]. The rate of utilization also appears not to affect measures of fatigue [90,91], however the kinetics and characteristics of the reconstitution of W′ are less understood.

Morton and Billat [92], first characterized intermittent exercise above and below CP as consisting of four parameters: work intensity above CP; work duration above CP; recovery intensity below CP; recovery duration below CP. Manipulating these parameters demonstrated that total work above CP can be increased beyond that achieved with constant severe intensity exercise, and a mathematical model was produced based on the linear expenditure and reconstitution of W′ at equal rates relative to CP [92]. Having a recovery rate identical to the expenditure rate is unlikely, and the reconstitution kinetics were later shown to be curvilinear with respect to recovery intensity [93], and recovery durations [94]. An investigation of the recovery of W′ at 2, 6 and 15 min demonstrated W′ reconstitution of 37%, 65% and 86%, respectively, when recovering at a nominal 20 W, although the authors chose not to describe the W′ reconstitution profile as anything more definitive than “curvilinear” on account of having only three data points. However, W′ reconstitution was quantified with a half time of 234 s, which was notably slower than that of $\dot{V}O$_2_ recovery kinetics (half time = 74 s) and faster than that of blood lactate kinetics (half time = 1366 s) [94]. Like W′, intramuscular PCr depletes when exercise is performed above CP [20], and increases when workload is reduced below CP [80]. That the kinetics of W′ reconstitution and $\dot{V}O$_2_ recovery (measured as a proxy for intramuscular phosphocreatine (PCr) reconstitution, [95]) did not align was suggestive of a process that may be partially dependent upon PCr reconstitution. Examining PCr levels during intermittent single leg exercise above and below CP revealed that the exhaustion of W′ coincided with the same level of PCr depletion regardless of recovery duration and that PCr reconstitution slowed with progressive bouts of intermittent exercise [96]. However, the intermittent protocol used in the study cannot determine W′ during the exercise and, therefore, cannot detect if W′ reconstitution itself slowed alongside that of PCr reconstitution.

Extending the mathematical CP model to include the reconstitution of W′ is extremely challenging. Unlike W′ expenditure, the reconstitution is not linear and is interdependent upon both recovery intensity and duration [93,94]. Furthermore, despite W′ being associated with thigh muscle size [97,98], $\dot{V}O$_2max_ [99] and the magnitude of the delta between $\dot{V}O$_2max_ and CP [99,100], the underlying physiological determinants of W′ remain largely unknown, necessitating that modelling be conducted on experimental data. Such a model was developed by Skiba, et al. [101], derived from an intermittent cycling protocol of 60 s severe intensity exercise and 30 s at different recovery intensities over four trials. Knowing the initial W′ and assuming exhaustion occurred when W′ was fully depleted, an integration model (henceforth referred to as Skiba1) was developed to predict the W′ balance (Equation (5)).

(5) $${W^{\prime }}_{\mathrm{bal}}\;=W^{\prime }-\overset{t}{\underset{0}{\int }}\left({W^{\prime }}_{\exp}\right)\times \left(e^{-\left(t-u\right)/\tau_{W^{\prime }}}\right)\times \mathrm{du}$$

where W′_bal_ = balance of W′ at time t (J); W′ = initial known W′ (J); W′_exp_ = total W′ expended (J); t − u = recovery duration (s); τ_W′_ = W′ reconstitution time constant (s).

The time constant (τ_W′_) was inversely related to CP indicating that those with the highest CP had the faster W′ reconstitution rates. An exponential regression yielded a method of determining τ_W′_ based on CP (Equation (6)).

τ_W′_ = 546 × e^(−0.01 D_CP_)^ + 316 (6)

where τ_W″_ = W′ reconstitution time constant (s); D_CP_ = Difference between the known CP and recovery power (W).

Equation (6) suggests a value of 316 W for difference between recovery power and CP (D_CP_) beyond which no further advantage is gained during recovery. As only one of the untrained participants had a CP in excess of this figure its validity remains theoretical. The experimental data from which the model was derived manipulated recovery intensity, but not duration. In a follow-up study [102], recovery and work durations varied between 5–20 s and 20–60 s, respectively, however the model underestimated W′ reconstitution in all work-recovery permutations, with the 60 s work and 30 s recovery as used in the original experiment producing the closest match to the predicted outcome. It was also noted that τ_W′_, as fitted to the experimental data, varied considerably between individuals possibly due to different muscle-fibre type composition, along with the recommendation that τ_W′_ be personalized for well-trained cyclists. To validate the Skiba1, the model was retrospectively applied to power data from a single race session, with the competitor abandoning the race when predicted W′_bal_ was approximately 1.5 kJ [101]. A further validation study using receiver operator curve analysis to separate signal from noise determined that exhaustion defined as a W′ balance of 1.5 kJ appropriately classified 80% of athletes as exhausted [103]. As the study did not actually necessitate participants to ride to absolute exhaustion, a balance of 1.5 kJ may be an appropriate threshold for the continuation of racing and training.

The Skiba1 model has inherent mathematical limitations such as W′ reconstitution whilst W′ is being expended and a reported imbalance of units [104]. Perhaps the biggest limitation of Skiba1, however, is that the recovery intensity can only be calculated retrospectively as it is averaged over a recovery interval. In order to overcome these limitations a differential model was derived (henceforth known as Skiba2) using the principles of chemical kinetics, with compartmentalized equations for power output above CP (Equation (7)) and below CP (Equation (8)) [105]. The Skiba2 model allows reconstitution to be calculated continuously, ultimately allowing a cyclist to view an estimation of their current W′ balance in real time assuming τ_W′_ has been individually and accurately calculated beforehand.

W′_bal_ = W′ − [(P − CP) × t] (7)

W′_bal_ = W′ − W′_exp_ × e^(D_CP_−t/τ_W′_)^ (8)

where W′_bal_ = balance of W′ at time t (J); W′ = W′ at start of bout (J); W′_exp_ = W′ expended (J); τ_W″_ = W′ reconstitution time constant (s); t = time (s); D_CP_ = difference between the known CP and recovery power (W).

The Skiba1 model was tested successfully against the “duty” cycle of handgrip tension and relaxation acting as a proxy for loading and unloading the leg whilst cycling, albeit with relaxation phases approximately six times that of cycling at a cadence of 90 r·min^−1^ [106], and the original 60/30 s intermittent cycling protocol was used to successfully validate the model in hypoxia on the condition that CP and W′ parameters were determined at the same hypoxic levels [107]. The application of cycling in hypoxia (such as when ascending a mountain) has been further considered resulting in a prediction equation for CP and W′ at altitude. This was tested using intermittent cycling against both Skiba models, with the Skiba2 model producing the closest match at the point of exhaustion [56]. Notably, both Skiba models were developed following experimental testing of untrained participants with τ_W′_ observed to be highly variable between participants [101,105]. The validity of the Skiba2 model for W′ reconstituted was assessed against elite athletes and was found to significantly underestimate their W′ reconstitution during intermittent exercise, resulting in the production of a modified τ_W′_ (Equation (9)) for this athletic population [108]. However, the testing of elite athletes warranted several methodological compromises in establishing CP and W′ parameters from training data rather than exhaustive tests, and recovery between trials was only 20 min. Thus, W′ was unlikely to have fully recovered. Like the Skiba models, the proposed τ_W′_ was reliant solely upon D_CP,_ yet the authors noted that increased aerobic fitness was likely to influence W′ reconstitution rates and that greater excess post-oxygen consumption was a probable mechanism for faster recovery [108,109]. A summary of W′ reconstitution studies can be seen in Table 1.

τ_W′_ = 2287.2 × D_CP_^−0.688^ (9)

where τ_W″_ = W′ reconstitution time constant (s); D_CP_ = difference between the known CP and recovery power (W).

The intermittent protocols used to develop and refine the models [56,101,108] rely upon only two known values of W′; a known individual W′ at the outset, and W′ of 0 J at exhaustion. The modelling is then built around a mono-exponential reconstitution of W′ throughout the intermittent bouts such that the two known values are satisfied with no known intermediate values, and no accounting for the potential slowing of W′ reconstitution as has been shown with PCr reconstitution [96]. A repeated ramp test to exhaustion allowed for the measurement of partial reconstitution of W′ after two minutes’ recovery and evidenced such a slowing of W′ reconstitution following successive bouts in both recreational and well-trained cyclists [45]. The ramp protocol employed also showed that the Skiba1 model did not fit well against the actual values of W′ determined from the test, overestimating W′ at the points of exhaustion and underestimating the rates of W′ reconstitution during recovery. Likewise, the Skiba1 model also underestimated W′ reconstitution after two minutes of recovery following CWR protocols, with modelled and actual values converging by six minutes of recovery [45]. Uniquely, Caen, et al. [110], found faster rates of W′ reconstitution following the faster depletion of W′ despite no differences in $\dot{V}O$_2_, blood lactate or pH measurements at the end of the depleting bout, further complicating the understanding of W′ reconstitution. Despite the criticisms of the Skiba models [45,104,108,110], they remain the only examples published to date.

A recent study investigating the physiological and anthropometric characteristics associated with the rate of W′ reconstitution [99], revealed that measures of aerobic fitness were related to W′ reconstitution, particularly in trained cyclists. Whilst CP was confirmed as having a positive relationship with W′ (as per the Skiba1 model for τ_W′_), a stronger relationship with $\dot{V}O$_2max_ existed for W′ reconstitution, along with relationships with both heart rate recovery and excess post-exercise oxygen consumption (EPOC). Relationships were also found between W′ reconstitution and fat mass in both trained and untrained subsets, suggesting a detrimental effect on W′ reconstitution. The slowing of W′ reconstitution following an exhaustive second bout was also inversely related to measures of $\dot{V}O$_2max_, heart rate recovery and EPOC in the trained subset only. Further insight into the quantification of the influence of these characteristics and their interdependency with training status is needed before models are produced with improved accuracy that can influence race performance (tactics) in real time.

## 5. The Application of Critical Power and W′ for Training Prescription

The field of sports science has developed numerous performance and physiological tests and measurements that are applicable to endurance sport and cycling [112,113,114]. However, for a variety of reasons none have become a standard measure for tracking physiological-related performance changes or prescribing training. Laboratory testing can be expensive, difficult to access, and intrusive to training for amateur and professional athletes alike. Also, step increments in test protocols, typically around 20–30 W [114,115,116], are too large to detect the differences in trained athletes where only small changes in performance measures are expected [117,118,119]. Accordingly, whilst few cyclists will know their $\dot{V}O$_2max_ or lactate thresholds, they will know their “functional threshold power” (FTP); a notional threshold power output that is sustainable for one hour [120], which, as a direct measure of performance, is accessible to any cyclist with a commercially available power meter. FTP is described as both analogous to lactate threshold, and similar to critical power in terms of fatigue occurring above the threshold power output [120], despite the physiological differences between these measures. The adoption of FTP as the de facto standard for performance measurement and tracking including amongst professional cyclists [121] has led to recent investigations into its physiological basis. As a measure of endurance performance FTP correlates strongly against other such endurance measures [122,123,124,125,126,127,128], but was not found to be an interchangeable or surrogate measure of lactate threshold [122,125,126,129,130], CP [131], respiratory compensation point [132], or MLSS [124,133] (see Table 2). This is unsurprising given that FTP is a measure of performance over an arbitrary chosen one-hour duration, which sits unquestionably within the confines of the heavy intensity domain, and as such does not align to any known physiological markers, thresholds or boundaries which define such laboratory-derived measurements [13]. Furthermore, to avoid the psychological factors that can affect fatigue over a 60-min trial, FTP is almost always estimated from a shorter 20-min test by scaling average power to 95% [120,121,122,123,124,125,126,129,134], or even an 8- min test scaling average power to 90% [129,130]. Thus, whilst FTP being in the heavy intensity domain will not deplete W′, the value is estimated from test protocols in the severe domain which expend an unknown amount of W′, further questioning its validity as either a performance measure or physiological proxy.

Despite the shortcomings of FTP, it is almost universally used for cycle training prescription. Market leading training software such as Training Peaks, Today’s Plan, Garmin Connect, and Zwift all use FTP as the single threshold marker, with training sessions based on percentages of FTP and training load calculations derived from it. To further compound the lack of validity for training prescription, numerous training zone schemas exist based upon arbitrary percentages of FTP, and as such these indiscriminate zones are physiologically meaningless. Training prescription based on FTP or zones derived from any single marker can yield quite different training responses in individuals owing to different relative intensities associated with the physiological intensity domains [135,136]. Using the parameters of the CP model can help reverse the disconnect between current training prescription methods and physiological responses to training. Rather than using arbitrary zones, training prescription should be based on intensity domains which elicit the desired physiological response. CP marks the boundary between heavy and severe domains and can be derived from laboratory or field tests described above, whilst the demarcation of the moderate and heavy intensity domains at lactate threshold can be estimated at 76% of CP [137]. However, more precise determination of either the lactate threshold or GET currently requires either analysis of blood samples or expired air, respectively [9]. Exercise tolerance within the severe intensity domain is determined by CP and W′ (Equation (1)). As W′ is not correlated to CP [74,101,103,138], simply specifying intensity and duration of severe intensity exercise based on principally aerobic measures of FTP or CP could lead to very different relative training sessions. For example, cyclist “A” has body mass = 78 kg; CP = 375 W; W′ = 13 kJ, and cyclist “B” has body mass = 64 kg; CP = 305 W; W′ = 13 kJ, meaning both cyclists have the same power to weight ratio and the same W′. A training session including 3-min at 120% of CP, would leave cyclist “B” with over 2 kJ remaining and so can continue the session [103], whilst cyclist “A” would fail to complete the effort due to exhaustion and the premature expenditure of W′. Constructing training from CP and W′ would allow relative intensities to be prescribed such that a fixed proportion of W′ is used within severe intensity exercise. In the above example, reducing cyclist B’s power output from 450 W to 436 W would produce the same 2 kJ (or 15%) of W′ remaining at the end of the effort.

The efficacy of high-intensity interval training in promoting performance improvements in cyclists is well established [139,140,141], however optimization of such training for individuals is impossible using current “threshold” prescription methods due to the lack W′ inclusion and the individual variability of the rate of W′ reconstitution [101,105]. The Skiba2 model can be used to monitor depletion of W′ in real time and assist in the execution of training; however, further work in refining the model is required in order to better predict W′ reconstitution kinetics so as to overcome the current limitations concerning applicability to differing protocols and progressive fatigue, before it can be used to optimize interval training. Should more precise models of W′ reconstitution become available, their usage could extend beyond training to race tactics and influence decisions about the rate and frequency of severe intensity attacks in bunch races, or the changeover order and work rates within team pursuits.

## 6. Conclusions

The two-parameter CP model mathematically describes the portion of the human power-duration relationship within a time frame constrained by the time taken for predominantly aerobic metabolism to reach CP, until the onset of fatigue when the maximum steady state can no longer be maintained. The CP parameter represents the power output associated with the maximum steady state and W′ the fixed quantity of work that can be performed within the severe intensity domain without any recovery and subsequent reconstitution of W′. Whilst other physiological measurements and arbitrary field measurements may be highly correlated to CP, CP alone marks the boundary of the heavy and severe intensity domains, either side of which elicit quite different physiological responses. Together CP and W′ have proven to be robust measurements allowing cyclists to predict their tolerable duration and power output within the severe intensity domain. The measurement of CP and W′ can be achieved either in the laboratory or in the field via multiple CWR trials, the 3-min all-out test, or a ramp all-out test, and whilst the CWR method may be the original research technique, it is the single test protocols that offer huge advantages to athletes in terms of practicalities. All methods of CP testing are extremely demanding and will induce a great deal of discomfort to the cyclist, and as such being “highly motivated” is unlikely to be sufficient to entice the athlete to reach their physiological limits needed to yield true measures of CP and W′. Therefore, consideration should be given to which protocol will help the individual cyclist to best approach those limits. The ramp all-out test arguably offers advantages in this respect due to the smooth ramp protocol, lack of conscious choice over when to end the ramp and the relatively short duration of the time in “pain”.

That CP marks a distinct physiological boundary and is measurable in the field, supports its use as the ideal candidate upon which to prescribe training and assess training load, for both amateur and professional cyclists, especially when compared to the standard FTP measurement which is designated as an arbitrary 60-min point on the power-duration relationship sitting well away from any physiological measurement. Severe intensity training is a mainstay of endurance training programmes, with the duration and intensity of tolerable work being dictated by W′ in addition to CP. Prescription of such training as a proportion of FTP or CP alone simply does not work owing to the lack of proportionality between such measurements and W′. Instead, basing severe intensity work on the fractional usage of W′ allows training to be planned and executed with much greater precision in order to meet desired training outcomes. High-intensity interval training cannot be optimized or prescribed individually using current techniques; however, modelling of W′ reconstitution would allow both the work and recovery phases of such sessions to be built into specific plans by athletes and coaches. The Skiba models demonstrate how this can be achieved with the addition of a single Tau parameter; however, it appears that W′ reconstitution is a complex matter, involving considerable individual variability and a slowing effect following repeated efforts, necessitating the validation of models against different protocols and with heterogeneous groups of cyclists. Nonetheless, while additional research is needed to improve the W′ reconstitution models, it is possible that training prescription could be better defined, and race plans based on knowledge of the reconstitution and expenditure of W′.

## Figures and Tables

**Table 1:** Summary of W′ reconstitution studies.

Study	Participants	Protocol Description *	Model ^†^	Principal Findings in Relation to W′ Reconstitution
Bartram, et al. [108]	4 male; elite cyclists	Intermittent: 30 s work/60 s recovery + open ended severe to finish	Skiba2	Skiba2 underestimated W′ reconstitution. New W′Tau formula proposed for elite cyclists
Broxterman, et al. [106]	6 male	Handgrip repetitions. Tension/relaxation of 50% and 20% duty cycles	Skiba1	Validated the Skiba1 model over a duty cycle which authors suggested as a proxy for the contraction and relaxation during a pedal revolution.
Caen, et al. [110]	11 male; physical exercise (PE) students	12 trials: 4 and 8 min exhaustive bouts, with 2,4,6 min recoveries	Skiba1	Skiba1 underestimated W′ reconstitution more so at 2-min recovery, less so at 6 min. Large individual variations in W′Tau. W′ reconstitution affected by preceding depletion rate, slower depletion = less reconstitution
Chidnok, et al. [93]	7 male; recreationally active	Intermittent: 60 s work/30 s recovery in differing domains	n/a	No recovery in severe domain. Recovery rate of W′ slower than expenditure in relation to critical power (CP).
Chidnok, et al. [96]	9 male; recreationally active	Single leg knee extensions. intermittent 60 s work/18, 30, 48 s recovery	n/a	W′ reconstitution increases with recovery duration. Phosphocreatine and pH levels were always the same at exhaustion. Phosphocreatine recovery correlated to W′ reconstitution but was faster. Phosphocreatine recovery slowed as exercise session progressed.
Chorley, et al. [45]	20 (19 male, 1 female; 9 trained, 11 untrained)	Repeated ramps to exhaustion with 2 min recoveries	Skiba1	Skiba1 did not fit the protocol, overestimate W′ at exhaustion and underestimating reconstitution during recoveries. W′ reconstitution slowed with repeated bouts of exhaustive exercise.
Chorley, et al. [99]	20 male; (9 trained, 11 untrained)	Repeated ramps to exhaustion with 2 min recoveries	Skiba1	Assessment of anthropometric and physiological relationships with W′ reconstitution and its slowing following repeated bouts.
Felippe, et al. [50]	10 male; recreationally active	2 × 6 min constant work rate (CWR) exhaustive bouts separated by 3, 6, 15 min recoveries	n/a	W′ reconstitution compared with neuromuscular recovery. Recovery of voluntary activation faster than W′, no difference between time constants of W′ and maximal voluntary contraction.
Ferguson, et al. [94]	6 male; recreationally active	6 min CWR exhaustive bout then 3, 6, 15 min recoveries	n/a	W′ reconstitution found to be curvilinear. Half-time of W′ reconstitution was faster than that of blood lactate but slower than that of oxygen uptake (a proxy for phosphocreatine reconstitution)
Morton and Billat [92]	6 male; well trained	Running: intermittent 60, 180, 30 s work, 60, 180 s recovery	n/a	Produced original model of linear W′ reconstitution at same rate as expenditure in relation to CP. Established W′ reconstitution occurred during recovery due to extended distances covered.
Shearman, et al. [107]	11 male; well trained	Intermittent: 60 s work/30 s recovery	Skiba1	Validated skiba1 in hypoxia with proviso that CP and W′ were also measured at same level of hypoxia
Skiba, et al. [101]	7 male; recreationally active	Intermittent: 60 s work/30 s recovery	Skiba1	Creation of Skiba1 W′bal model based on intermittent exercise to exhaustion, together with generic Tau equation based on CP. Validated against single rider in a race with W′bal of 1.5 kJ at retirement from race.
Skiba, et al. [103]	8 (6 male, 2 female) 8 well trained triathletes	Assessment of training and race data	Skiba1	Validation of Skiba1 on training and race data to detect the point of exhaustion. When exhaustion is set at W′bal = 1.5 kJ prediction of exhaustion was 80% appropriately classified as exhausted and 88% appropriately classified as non-exhausted. Recommendation to use 1.5 kJ as practical level of exhaustion.
Skiba, et al. [105]	10 (6 male, 4 female); recreationally active	Intermittent: 60, 40, 20 s work/30, 20, 10, 5 s recovery	Skiba1	Skiba1 underestimated W′ reconstitution, more so with reduced work and/or recovery durations. Large individual variations in reconstitution rate hence recommendations to individualize Tau.
Skiba, et al. [102]	11 (5 male, 6 female); recreationally active	Cycle and single leg knee extensions. 3 min CWR exhaustive bout then 1, 2, 5, 7 min recoveries	Skiba2	Skiba2 differential model produced allowing real time W′bal prediction. Large inter and intra individual variations in reconstitution rate observed.
Sreedhara, et al. [104]	7 male; trained	120 s bout to deplete 50% of W′, followed by 2, 6, 15 min recoveries, followed by 3-min all-out	Skiba2	Skiba2 overestimated W′ reconstitution, based on the estimated 50% of W′ expended during initial bout. W′ reconstitution did not increase from 6 min to 15 min recovery hence W′ reconstitution was not exponential.
Townsend, et al. [56]	9 male; trained	Intermittent: 40–60 s work/30–60 s recovery	Skiba1 and Skiba2	Produced a modification equation for CP based on altitude for use in Skiba models to allow W′ reconstitution to be predicted at increasing altitude.
Vanhatalo and Jones [40]	7 male; recreationally active	30 s sprint, followed by 2- or 15- min recovery then 3-min all-out test	n/a	Prior severe sprint exercise (extent of W′ expenditure unknown) depletes W′ but not CP. W′ reconstruction of 79% after 2 min and fully recovered by 15 min
Vinetti, et al. [111]	7 male; recreationally active	Incremental ramp with steps 30–300 s duration with recovery between each step of 0–180 s.	n/a	Extensive mathematical representation of discontinuous ramp exercise.

* Study protocols are cycling based unless specified otherwise. ^†^ The W′ reconstitution model referenced or assessed in the study where Skiba1 is the integration model (Equation (5)) and Skiba2 is the differential model (Equations (7) and (8)).

**Table 2:** Comparisons of functional threshold power to previously validated physiological measurements.

Study	Participants	Functional Threshold Power Test Method	Validated Against *	Mean Functional Threshold Power (W)	Comparison Mean Power Output (W)	Significantly Different	Correlation Coefficient (r)	Comments
Barranco-Gil, et al. [132]	15 male, well trained	20-min test	RCP	284 to 286	344 ± 32	Yes	0.86 to 0.93	Range of FTP and correlation coefficients due to 3 warm up techniques providing Functional Threshold Power (FTP) values of 286 ± 26 W; 284 ± 26 W; 286 ± 32 W
Borszcz, et al. [122]	23 male, trained	20-min test	IAT	236 ± 38	344 ± 32	No	0.61	Graded test with large 40 W increments used to determine IAT
		60-min test	IAT	231 ± 33	237 ± 29	No	0.76	
Gavin, et al. [129]	7 male, trained and well trained	8-min test	OBLA	301 ± 13	293 ± 9	No (see notes)	0.70	OBLA selected from three other Lactate measurements as most appropriate comparison for FTP
Inglis, et al. [124]	18 (12 male 6 female), trained and well trained	20-min test	MLSS	261 ± 45	243 ± 48	Yes	0.96	
Jeffries, et al. [125]	20 male, well trained	20-min test	LT (Dmax)	266 ± 42	221 ± 25	Yes	0.80	
			LT (modified Dmax)	266 ± 42	238 ± 32	Yes	0.75	
			OBLA	266 ± 42	268 ± 30	No	0.88	authors noted that despite no significant difference between FTP and OBLA, large random error made in individual data meant that FTP was not equivalent to OBLA
			IAT	266 ± 42	244 ± 33	Yes	0.85	
Klitzke Borszcz, et al. [123]	15 male, trained and well trained	20-min test	MLSS	252 ± 23	248 ± 25	No	0.91	Nine out of 12 participants had difference between MLSS and FTP of 5% or more
Lillo-Bevia, et al. [133]	11 male, trained	20-min test	MLSS	262 ± 19	250 ± 16	Yes	0.95	
MacInnis, et al. [127]	8 male, well trained	60-min test	CP	309 ± 26	325 ± 29	Yes	0.91	Critical power derived from a 4-min and 20-min test, the latter of which is longer than generally accepted for CP testing.
McGrath, et al. [128]	19 (12 male 7 female) well trained	20-min test	LT (Dmax)	259 ± 40	246 ± 38	Not reported	0.94	authors noted large limits of agreement meaning that FTP was not equivalent to Lactate threshold
Morgan, et al. [131]	12 male, trained	20-min test	LT (Dmax)	278 ± 42	275 ± 40	No	0.92	authors noted that despite no significant difference between FTP and CP, large limits of agreement meant that FTP was not equivalent to CP
Sanders, et al. [130]	19 male, well trained	8-min test	LT (DMax)	341 ± 33	279 ± 20	Very largely different	Not reported	
			LT (modified Dmax)	341 ± 33	319 ± 29	Moderately different	Not reported	
			OBLA	341 ± 33	319 ± 25	Moderately different	Not reported	
Valenzuela, et al. [126]	20 male, cyclists	20-min test	LT (modified Dmax)	240 ± 35	246 ± 24	No	0.90	
	Subset: 11 recreational cyclists			≈217	≈232	Yes	0.88	subgroup power outputs are derived from mean body mass × w/kg for each subgroup as FTP and LT subgroup means are not quoted in the study.
	Subset: 9 well trained cyclists			≈269 W	≈265	No	095	

* Respiratory compensation point (RCP); individual anaerobic threshold (IAT); onset of blood lactate accumulation (OBLA); maximal lactate steady state (MLSS); lactate threshold (LT); critical power (CP).

## References

[1] Hill A.V. (1925). The physiological basis of athletic records. Nature.

[2] Monod H., Scherrer J. (1965). The work capacity of a synergic muscular group. Ergonomics.

[3] Moritani T., Nagata A., Devries H.A., Muro M. (1981). Critical power as a measure of physical work capacity and anaerobic threshold. Ergonomics.

[4] Poole D.C., Ward S.A., Gardner G.W., Whipp B.J. (1988). Metabolic and respiratory profile of the upper limit for prolonged exercise in man. Ergonomics.

[5] Davis H.A., Bassett J., Hughes P., Gass G.C. (1983). Anaerobic threshold and lactate turnpoint. Eur. J. Appl. Physiol. Occup. Physiol..

[6] Cheng B., Kuipers H., Snyder A.C., Keizer H.A., Jeukendrup A., Hesselink M. (1992). A new approach for the determination of ventilatory and lactate thresholds. Int. J. Sports Med..

[7] Tanaka K., Matsuura Y., Kumagai S., Matsuzaka A., Hirakoba K., Asano K. (1983). Relationships of anaerobic threshold and onset of blood lactate accumulation with endurance performance. Eur. J. Appl. Physiol. Occup. Physiol..

[8] Beneke R., von Duvillard S.P. (1996). Determination of maximal lactate steady state response in selected sports events. Med. Sci. Sports Exerc..

[9] Beaver W.L., Wasserman K., Whipp B.J. (1986). A new method for detecting anaerobic threshold by gas-exchange. J. Appl. Physiol..

[10] Simon J., Young J.L., Gutin B., Blood D.K., Case R.B. (1983). Lactate accumulation relative to the anaerobic and respiratory compensation thresholds. J. Appl. Physiol. Respir. Environ. Exerc. Physiol..

[11] Hill D.W. (1993). The critical power concept—A review. Sports Med..

[12] Burnley M., Doust J.H., Vanhatalo A. (2006). A 3-min all-out test to determine peak oxygen uptake and the maximal steady state. Med. Sci. Sports Exerc..

[13] Jones A.M., Burnley M., Black M.I., Poole D.C., Vanhatalo A. (2019). The maximal metabolic steady state: Redefining the “gold standard”. Physiol. Rep..

[14] Mitchell E.A., Martin N.R.W., Bailey S.J., Ferguson R.A. (2018). Critical power is positively related to skeletal muscle capillarity and type i muscle fibers in endurance-trained individuals. J. Appl. Physiol..

[15] Hill D.W., Smith J.C. (1993). A comparison of methods of estimating anaerobic work capacity. Ergonomics.

[16] Miura A., Sato H., Sato H., Whipp B.J., Fukuba Y. (2000). The effect of glycogen depletion on the curvature constant parameter of the power-duration curve for cycle ergometry. Ergonomics.

[17] Valli G., Cogo A., Passino C., Bonardi D., Morici G., Fasano V., Agnesi M., Bernardi L., Ferrazza A.M., Ward S.A. (2011). Exercise intolerance at high altitude (5050 m): Critical power and w′. Respir. Physiol. Neurobiol..

[18] Simpson L.P., Jones A.M., Skiba P.F., Vanhatalo A., Wilkerson D. (2015). Influence of hypoxia on the power-duration relationship during high-intensity exercise. Int. J. Sports Med..

[19] Burnley M., Jones A.M. (2007). Oxygen uptake kinetics as a determinant of sports performance. Eur. J. Sport Sci..

[20] Jones A.M., Wilkerson D.P., DiMenna F., Fulford J., Poole D.C. (2008). Muscle metabolic responses to exercise above and below the “critical power” assessed using 31p-mrs. Am. J. Physiol. Regul. Integr. Comp. Physiol..

[21] Fukuba Y., Miura A., Endo M., Kan A., Yanagawa K., Whipp B.J. (2003). The curvature constant parameter of the power-duration curve for varied-power exercise. Med. Sci. Sports Exerc..

[22] Poole D.C., Burnley M., Vanhatalo A., Rossiter H.B., Jones A.M. (2016). Critical power: An important fatigue threshold in exercise physiology. Med. Sci. Sports Exerc..

[23] Johnson M.A., Mills D.E., Brown P.I., Sharpe G.R. (2014). Prior upper body exercise reduces cycling work capacity but not critical power. Med. Sci. Sports Exerc..

[24] Morton R.H. (2006). The critical power and related whole-body bioenergetic models. Eur. J. Appl. Physiol..

[25] Pringle J.S.M., Jones A.M. (2002). Maximal lactate steady state, critical power and emg during cycling. Eur. J. Appl. Physiol..

[26] Brickley G., Doust J., Williams C.A. (2002). Physiological responses during exercise to exhaustion at critical power. Eur. J. Appl. Physiol..

[27] Hill D.W., Smith J.C. (1999). Determination of critical power by pulmonary gas exchange. Can. J. Appl. Physiol..

[28] Clark I.E., Vanhatalo A., Thompson C., Joseph C., Black M.I., Blackwell J.R., Wylie L.J., Tan R., Bailey S.J., Wilkins B.W. (2019). Dynamics of the power-duration relationship during prolonged endurance exercise and influence of carbohydrate ingestion. J. Appl. Physiol..

[29] Jeukendrup A.E., Wallis G.A. (2005). Measurement of substrate oxidation during exercise by means of gas exchange measurements. Int. J. Sports Med..

[30] Morton R.H. (1990). Modelling human power and endurance. J. Math. Biol..

[31] Morton R.H. (1996). A 3-parameter critical power model. Ergonomics.

[32] Gaesser G.A., Carnevale T.J., Garfinkel A., Walter D.O., Womack C.J. (1995). Estimation of critical power with nonlinear and linear-models. Med. Sci. Sports Exerc..

[33] Bergstrom H.C., Housh T.J., Zuniga J.M., Traylor D.A., Lewis R.W., Camic C.L., Schmidt R.J., Johnson G.O. (2014). Differences among estimates of critical power and anaerobic work capacity derived from five mathematical models and the three-minute all-out test. J. Strength Cond. Res..

[34] Di Prampero P.E. (1999). The concept of critical velocity: A brief analysis. Eur. J. Appl. Physiol. Occup. Physiol..

[35] Burnley M., Davison G., Baker J.R. (2011). Effects of priming exercise on vo2 kinetics and the power-duration relationship. Med. Sci. Sports Exerc..

[36] Bartram J.C., Thewlis D., Martin D.T., Norton K.I. (2017). Predicting critical power in elite cyclists: Questioning the validity of the 3-minute all-out test. Int. J. Sports Physiol. Perform..

[37] Jones A.M., Vanhatalo A., Burnley M., Morton R.H., Poole D.C. (2010). Critical power: Implications for determination of vo2max and exercise tolerance. Med. Sci. Sports Exerc..

[38] Vanhatalo A., Jones A.M., Burnley M. (2011). Application of critical power in sport. Int. J. Sports Physiol. Perform..

[39] Muniz-Pumares D., Pedlar C., Godfrey R., Glaister M. (2017). A comparison of methods to estimate anaerobic capacity: Accumulated oxygen deficit and w′ during constant and all-out work-rate profiles. J. Sports Sci..

[40] Vanhatalo A., Jones A.M. (2009). Influence of prior sprint exercise on the parameters of the “all-out critical power test” in men. Exp. Physiol..

[41] Marcora S.M., Staiano W. (2010). The limit to exercise tolerance in humans: Mind over muscle?. Eur. J. Appl. Physiol..

[42] Salam H., Marcora S.M., Hopker J.G. (2018). The effect of mental fatigue on critical power during cycling exercise. Eur. J. Appl. Physiol..

[43] Staiano W., Bosio A., Morree H.M., Rampinini E., Marcora S. (2018). The cardinal exercise stopper: Muscle fatigue, muscle pain or perception of effort?. Prog. Brain Res..

[44] Gaesser G.A., Wilson L.A. (1988). Effects of continuous and interval training on the parameters of the power-endurance time relationship for high-intensity exercise. Int. J. Sports Med..

[45] Chorley A., Bott R.P., Marwood S., Lamb K.L. (2019). Slowing the reconstitution of w′ in recovery with repeated bouts of maximal exercise. Int. J. Sports Physiol. Perform..

[46] Chidnok W., Dimenna F.J., Bailey S.J., Wilkerson D.P., Vanhatalo A., Jones A.M. (2013). Effects of pacing strategy on work done above critical power during high-intensity exercise. Med. Sci. Sports Exerc..

[47] Murgatroyd S.R., Ferguson C., Ward S.A., Whipp B.J., Rossiter H.B. (2011). Pulmonary o-2 uptake kinetics as a determinant of high-intensity exercise tolerance in humans. J. Appl. Physiol..

[48] Murgatroyd S.R., Wylde L.A., Cannon D.T., Ward S.A., Rossiter H.B. (2014). A “ramp-sprint” protocol to characterise indices of aerobic function and exercise intensity domains in a single laboratory test. Eur. J. Appl. Physiol..

[49] Vanhatalo A., Doust J.H., Burnley M. (2007). Determination of critical power using a 3-min all-out cycling test. Med. Sci. Sports Exerc..

[50] Felippe L.C., Melo T.G., Silva-Cavalcante M.D., Ferreira G.A., Boari D., Bertuzzi R., Lima-Silva A.E. (2020). Relationship between recovery of neuromuscular function and subsequent capacity to work above critical power. Eur. J. Appl. Physiol..

[51] Yong S.E., Swisher A.R., Ferguson C., Cannon D.T. (2019). Maximal sustained isokinetic power at exercise intolerance is not critical power. Int. J. Sports Med..

[52] Mitchell E.A., Martin N.R.W., Turner M.C., Taylor C.W., Ferguson R.A. (2019). The combined effect of sprint interval training and postexercise blood flow restriction on critical power, capillary growth, and mitochondrial proteins in trained cyclists. J. Appl. Physiol..

[53] Muniz-Pumares D., Karsten B., Triska C., Glaister M. (2019). Methodological approaches and related challenges associated with the determination of critical power and curvature constant. J. Strength Cond. Res..

[54] Black M.I., Jones A.M., Bailey S.J., Vanhatalo A. (2015). Self-pacing increases critical power and improves performance during severe-intensity exercise. Appl. Physiol. Nutr. Metab..

[55] Black M.I., Jones A.M., Blackwell J.R., Bailey S.J., Wylie L.J., McDonagh S.T., Thompson C., Kelly J., Sumners P., Mileva K.N. (2017). Muscle metabolic and neuromuscular determinants of fatigue during cycling in different exercise intensity domains. J. Appl. Physiol..

[56] Townsend N.E., Nichols D.S., Skiba P.F., Racinais S., Periard J.D. (2017). Prediction of critical power and w′ in hypoxia: Application to work-balance modelling. Front. Physiol..

[57] Jeukendrup A., Saris W.H., Brouns F., Kester A.D. (1996). A new validated endurance performance test. Med. Sci. Sports Exerc..

[58] Currell K., Jeukendrup A.E. (2008). Validity, reliability and sensitivity of measures of sporting performance. Sports Med..

[59] Bishop D., Jenkins D.G., Howard A. (1998). The critical power function is dependent on the duration of the predictive exercise tests chosen. Int. J. Sports Med..

[60] Morton R.H. (1994). Critical power test for ramp exercise. Eur. J. Appl. Physiol. Occup. Physiol..

[61] Morton R.H., Green S., Bishop D., Jenkins D.G. (1997). Ramp and constant power trials produce equivalent critical power estimates. Med. Sci. Sports Exerc..

[62] Thomas K., Goodall S., Stone M., Howatson G., St Clair Gibson A., Ansley L. (2015). Central and peripheral fatigue in male cyclists after 4-, 20-, and 40-km time trials. Med. Sci. Sports Exerc..

[63] Karsten B., Jobson S.A., Hopker J., Jimenez A., Beedie C. (2014). High agreement between laboratory and field estimates of critical power in cycling. Int. J. Sports Med..

[64] Brickley G., Green S., Jenkins D.G., McEinery M., Wishart C., Doust J.D., Williams C.A. (2007). Muscle metabolism during constant- and alternating-intensity exercise around critical power. Int. J. Sports Med..

[65] Karsten B., Hopker J., Jobson S.A., Baker J., Petrigna L., Klose A., Beedie C. (2017). Comparison of inter-trial recovery times for the determination of critical power and w′ in cycling. J. Sports Sci..

[66] Karsten B., Jobson S.A., Hopker J., Stevens L., Beedie C. (2015). Validity and reliability of critical power field testing. Eur. J. Appl. Physiol..

[67] Vanhatalo A., Doust J.H., Burnley M. (2008). A 3-min all-out cycling test is sensitive to a change in critical power. Med. Sci. Sports Exerc..

[68] Wright J., Bruce-Low S., Jobson S.A. (2017). The reliability and validity of the 3-min all-out cycling critical power test. Int. J. Sports Med..

[69] Karsten B., Jobson S.A., Hopker J., Passfield L., Beedie C. (2014). The 3-min test does not provide a valid measure of critical power using the srm isokinetic mode. Int. J. Sports Med..

[70] Clark I.E., Murray S.R., Pettitt R.W. (2013). Alternative procedures for the three-minute all-out exercise test. J. Strength Cond. Res..

[71] Vanhatalo A., Doust J.H., Burnley M. (2008). Robustness of a 3 min all-out cycling test to manipulations of power profile and cadence in humans. Exp. Physiol..

[72] Black M.I., Durant J., Jones A.M., Vanhatalo A. (2014). Critical power derived from a 3-min all-out test predicts 16.1-km road time-trial performance. Eur. J. Sport Sci..

[73] Constantini K., Sabapathy S., Cross T.J. (2014). A single-session testing protocol to determine critical power and w′. Eur. J. Appl. Physiol..

[74] Bergstrom H.C., Housh T.J., Zuniga J.M., Camic C.L., Traylor D.A., Schmidt R.J., Johnson G.O. (2012). A new single work bout test to estimate critical power and anaerobic work capacity. J. Strength Cond. Res..

[75] Johnson T.M., Sexton P.J., Placek A.M., Murray S.R., Pettitt R.W. (2011). Reliability analysis of the 3-min all-out exercise test for cycle ergometry. Med. Sci. Sports Exerc..

[76] Dicks N.D., Jamnick N.A., Murray S.R., Pettitt R.W. (2016). Load determination for the 3-minute all-out exercise test for cycle ergometry. Int. J. Sports Physiol. Perform..

[77] Boone J., Bourgois J. (2012). The oxygen uptake response to incremental ramp exercise methodogical and physiological issues. Sports Med..

[78] Whipp B.J., Davis J.A., Torres F., Wasserman K. (1981). A test to determine parameters of aerobic function during exercise. J. Appl. Physiol. Respir. Environ. Exerc. Physiol..

[79] Black M.I., Jones A.M., Kelly J.A., Bailey S.J., Vanhatalo A. (2016). The constant work rate critical power protocol overestimates ramp incremental exercise performance. Eur. J. Appl. Physiol..

[80] Chidnok W., Fulford J., Bailey S.J., Dimenna F.J., Skiba P.F., Vanhatalo A., Jones A.M. (2013). Muscle metabolic determinants of exercise tolerance following exhaustion: Relationship to the “critical power”. J. Appl. Physiol..

[81] Coats E.M., Rossiter H.B., Day J.R., Miura A., Fukuba Y., Whipp B.J. (2003). Intensity-dependent tolerance to exercise after attaining vo2 max in humans. J. Appl. Physiol..

[82] Van der Vaart H., Murgatroyd S.R., Rossiter H.B., Chen C., Casaburi R., Porszasz J. (2014). Selecting constant work rates for endurance testing in copd: The role of the power-duration relationship. COPD J. Chronic Obstr. Pulm. Dis..

[83] Wright J., Walker T., Burnet S., Jobson S.A. (2019). The reliability and validity of the powertap p1 power pedals before and after 100 hours of use. Int. J. Sports Physiol. Perform..

[84] Nimmerichter A., Schnitzer L., Prinz B., Simon D., Wirth K. (2017). Validity and reliability of the garmin vector power meter in laboratory and field cycling. Int. J. Sports Med..

[85] Hopker J., Myers S., Jobson S.A., Bruce W., Passfield L. (2010). Validity and reliability of the wattbike cycle ergometer. Int. J. Sports Med..

[86] Whittle C., Smith N., Jobson S.A. (2018). Validity of powertap p1 pedals during laboratory-based cycling time trial performance. Sports.

[87] Jeukendrup A.E., Craig N.P., Hawley J.A. (2000). The bioenergetics of world class cycling. J. Sci. Med. Sport.

[88] Abbiss C.R., Laursen P.B. (2008). Describing and understanding pacing strategies during athletic competition. Sports Med..

[89] Abbiss C.R., Menaspa P., Villerius V., Martin D.T. (2013). Distribution of power output when establishing a breakaway in cycling. Int. J. Sports Physiol. Perform..

[90] De Souza K.M., Dekerle J., Salvador P.C.D., de Lucas R.D., Guglielmo L.G.A., Greco C.C., Denadai B.S. (2016). Rate of utilization of a given fraction of w′ (the curvature constant of the power-duration relationship) does not affect fatigue during severe-intensity exercise. Exp. Physiol..

[91] Burnley M., Vanhatalo A., Jones A.M. (2012). Distinct profiles of neuromuscular fatigue during muscle contractions below and above the critical torque in humans. J. Appl. Physiol..

[92] Morton R.H., Billat L.V. (2004). The critical power model for intermittent exercise. Eur. J. Appl. Physiol..

[93] Chidnok W., Dimenna F.J., Bailey S.J., Vanhatalo A., Morton R.H., Wilkerson D.P., Jones A.M. (2012). Exercise tolerance in intermittent cycling: Application of the critical power concept. Med. Sci. Sports Exerc..

[94] Ferguson C., Rossiter H.B., Whipp B.J., Cathcart A.J., Murgatroyd S.R., Ward S.A. (2010). Effect of recovery duration from prior exhaustive exercise on the parameters of the power-duration relationship. J. Appl. Physiol..

[95] Rossiter H.B., Ward S.A., Kowalchuk J.M., Howe F.A., Griffiths J.R., Whipp B.J. (2002). Dynamic asymmetry of phosphocreatine concentration and o-2 uptake between the on- and off-transients of moderate- and high-intensity exercise in humans. J. Physiol. Lond..

[96] Chidnok W., DiMenna F.J., Fulford J., Bailey S.J., Skiba P.F., Vanhatalo A., Jones A.M. (2013). Muscle metabolic responses during high-intensity intermittent exercise measured by (31)p-mrs: Relationship to the critical power concept. Am. J. Physiol. Regul. Integr. Comp. Physiol..

[97] Kordi M., Menzies C., Parker Simpson L. (2018). Relationship between power–duration parameters and mechanical and anthropometric properties of the thigh in elite cyclists. Eur. J. Appl. Physiol..

[98] Miura A., Endo M., Sato H., Sato H., Barstow T.J., Fukuba Y. (2002). Relationship between the curvature constant parameter of the power-duration curve and muscle cross-sectional area of the thigh for cycle ergometry in humans. Eur. J. Appl. Physiol..

[99] Chorley A., Bott R.P., Marwood S., Lamb K.L. (2020). Physiological and anthropometric determinants of critical power, w′ and the reconstitution of w′ in trained and untrained male cyclists. Eur. J. Appl. Physiol..

[100] Vanhatalo A., Poole D.C., DiMenna F.J., Bailey S.J., Jones A.M. (2011). Muscle fiber recruitment and the slow component of o2 uptake: Constant work rate vs. All-out sprint exercise. Am. J. Physiol. Regul. Integr. Comp. Physiol..

[101] Skiba P.F., Chidnok W., Vanhatalo A., Jones A.M. (2012). Modeling the expenditure and reconstitution of work capacity above critical power. Med. Sci. Sports Exerc..

[102] Skiba P.F., Jackman S., Clarke D., Vanhatalo A., Jones A.M. (2014). Effect of work and recovery durations on w′ reconstitution during intermittent exercise. Med. Sci. Sports Exerc..

[103] Skiba P.F., Clarke D., Vanhatalo A., Jones A.M. (2014). Validation of a novel intermittent w′ model for cycling using field data. Int. J. Sports Physiol. Perform..

[104] Sreedhara V.S.M., Ashtiani F., Mocko G.M., Vahidi A., Hutchison R.E. (2020). Modeling the recovery of w′ in the moderate to heavy exercise intensity domain. Med. Sci. Sports Exerc..

[105] Skiba P.F., Fulford J., Clarke D.C., Vanhatalo A., Jones A.M. (2015). Intramuscular determinants of the ability to recover work capacity above critical power. Eur. J. Appl. Physiol..

[106] Broxterman R.M., Skiba P.F., Craig J.C., Wilcox S.L., Ade C.J., Barstow T.J. (2016). w′ expenditure and reconstitution during severe intensity constant power exercise: Mechanistic insight into the determinants of w′. Respir. Physiol. Neurobiol..

[107] Shearman S., Dwyer D., Skiba P., Townsend N. (2016). Modeling intermittent cycling performance in hypoxia using the critical power concept. Med. Sci. Sports Exerc..

[108] Bartram J.C., Thewlis D., Martin D.T., Norton K.I. (2017). Accuracy of w′ recovery kinetics in high performance cyclists—Modelling intermittent work capacity. Int. J. Sports Physiol. Perform..

[109] Tomlin D.L., Wenger H.A. (2001). The relationship between aerobic fitness and recovery from high intensity intermittent exercise. Sports Med..

[110] Caen K., Bourgois J.G., Bourgois G., van Der Stede T., Vermeire K., Boone J. (2019). The reconstitution of w′ depends on both work and recovery characteristics. Med. Sci. Sports Exerc..

[111] Vinetti G., Fagoni N., Taboni A., Camelio S., di Prampero P.E., Ferretti G. (2017). Effects of recovery interval duration on the parameters of the critical power model for incremental exercise. Eur. J. Appl. Physiol..

[112] Hopkins S.R., McKenzie D.C. (1994). The laboratory assessment of endurance performance in cyclists. Can. J. Appl. Physiol..

[113] Zhou S., Robson S.J., King M.J., Davie A.J. (1997). Correlations between short-course triathlon performance and physiological variables determined in laboratory cycle and treadmill tests. J. Sports Med. Phys. Fit..

[114] Bishop D., Jenkins D.G., Mackinnon L.T. (1998). The relationship between plasma lactate parameters, wpeak and 1-h cycling performance in women. Med. Sci. Sports Exerc..

[115] Adami A., Sivieri A., Moia C., Perini R., Ferretti G. (2013). Effects of step duration in incremental ramp protocols on peak power and maximal oxygen consumption. Eur. J. Appl. Physiol..

[116] Bentley D.J., Newell J., Bishop D. (2007). Incremental exercise test design and analysis—Implications for performance diagnostics in endurance athletes. Sports Med..

[117] Paton C.D., Hopkins W.G. (2006). Variation in performance of elite cyclists from race to race. Eur. J. Sport Sci..

[118] Nimmerichter A., Eston R.G., Bachl N., Williams C. (2011). Longitudinal monitoring of power output and heart rate profiles in elite cyclists. J. Sports Sci..

[119] Hopkins W.G., Hawley J.A., Burke L.M. (1999). Design and analysis of research on sport performance enhancement. Med. Sci. Sports Exerc..

[120] Allen H., Coggan A. (2010). Training and Racing with a Power Meter.

[121] Van Erp T., Sanders D. (2020). Demands of professional cycling races: Influence of race category and result. Eur. J. Sport Sci..

[122] Borszcz F.K., Tramontin A.F., Bossi A.H., Carminatti L.J., Costa V.P. (2018). Functional threshold power in cyclists: Validity of the concept and physiological responses. Int. J. Sports Med..

[123] Klitzke Borszcz F., Ferreira Tramontin A., Pereira Costa V. (2019). Is the functional threshold power interchangeable with the maximal lactate steady state in trained cyclists?. Int. J. Sports Physiol. Perform..

[124] Inglis E.C., Iannetta D., Passfield L., Murias J.M. (2019). Maximal lactate steady state versus the 20-minute functional threshold power test in well-trained individuals: “Watts” the big deal?. Int. J. Sports Physiol. Perform..

[125] Jeffries O., Simmons R., Patterson S.D., Waldron M. (2019). Functional threshold power is not equivalent to lactate parameters in trained cyclists. J. Strength Cond. Res..

[126] Valenzuela P.L., Morales J.S., Foster C., Lucia A., de la Villa P. (2018). Is the functional threshold power a valid surrogate of the lactate threshold?. Int. J. Sports Physiol. Perform..

[127] MacInnis M.J., Thomas A.C.Q., Phillips S.M. (2019). The reliability of 4-minute and 20-minute time trials and their relationships to functional threshold power in trained cyclists. Int. J. Sports Physiol. Perform..

[128] McGrath E., Mahony N., Fleming N., Donne B. (2019). Is the ftp test a reliable, reproducible and functional assessment tool in highly-trained athletes?. Int. J. Exerc. Sci..

[129] Gavin T.P., Van Meter J.B., Brophy P.M., Dubis G.S., Potts K.N., Hickner R.C. (2012). Comparison of a field-based test to estimate functional threshold power and power output at lactate threshold. J. Strength Cond. Res..

[130] Sanders D., Taylor R.J., Myers T., Akubat I. (2017). A field-based cycling test to assess predictors of endurance performance and establishing training zones. J. Strength Cond. Res..

[131] Morgan P.T., Black M.I., Bailey S.J., Jones A.M., Vanhatalo A. (2019). Road cycle tt performance: Relationship to the power-duration model and association with ftp. J. Sports Sci..

[132] Barranco-Gil D., Gil-Cabrera J., Valenzuela P.L., Alejo L.B., Montalvo-Perez A., Talavera E., Moral-Gonzalez S., Lucia A. (2020). Functional threshold power: Relationship with respiratory compensation point and effects of various warm-up protocols. Int. J. Sports Physiol. Perform..

[133] Lillo-Bevia J.R., Courel-Ibanez J., Cerezuela-Espejo V., Moran-Navarro R., Martinez-Cava A., Pallares J.G. (2019). Is the functional threshold power a valid metric to estimate the maximal lactate steady state in cyclists?. J. Strength Cond. Res..

[134] Klitzke Borszcz F., Tramontin A.F., Costa V.P. (2020). Reliability of the functional threshold power in competitive cyclists. Int. J. Sports Med..

[135] Mann T., Lamberts R.P., Lambert M.I. (2013). Methods of prescribing relative exercise intensity: Physiological and practical considerations. Sports Med..

[136] Keir D.A., Fontana F.Y., Robertson T.C., Murias J.M., Paterson D.H., Kowalchuk J.M., Pogliaghi S. (2015). Exercise intensity thresholds: Identifying the boundaries of sustainable performance. Med. Sci. Sports Exerc..

[137] Froyd C., Millet G.Y., Noakes T.D. (2013). The development of peripheral fatigue and short-term recovery during self-paced high-intensity exercise. J. Physiol. Lond..

[138] Vanhatalo A., Jones A.M. (2009). Influence of creatine supplementation on the parameters of the “all-out critical power test”. J. Exerc. Sci. Fit..

[139] Ronnestad B.R., Hansen J., Ellefsen S. (2014). Block periodization of high-intensity aerobic intervals provides superior training effects in trained cyclists. Scand. J. Med. Sci. Sports.

[140] Milanovic Z., Sporis G., Weston M. (2015). Effectiveness of high-intensity interval training (hit) and continuous endurance training for vo2max improvements: A systematic review and meta-analysis of controlled trials. Sports Med..

[141] Laursen P.B., Jenkins D.G. (2002). The scientific basis for high-intensity interval training: Optimising training programmes and maximising performance in highly trained endurance athletes. Sports Med..

